# Fertility preservation is an imperative goal in the clinical practice of radiation oncology: a narrative review

**DOI:** 10.3332/ecancer.2022.1461

**Published:** 2022-11-02

**Authors:** Yumna Ahmed, Agha Muhammad Hammad Khan, Urooba Jawwad Rao, Fatima Shaukat, Arhum Jamil, Syed Mohammad Hasan, Sehrish Abrar, Bilal Mazhar Qureshi, Ahmed Nadeem Abbasi

**Affiliations:** 1 Department of Radiation Oncology, Aga Khan University Hospital, Stadium Road, P. O. Box 3500, Karachi 74800, Pakistan; 2 Sultan Qaboos Comprehensive Cancer Care and Research Centre, Seeb 123, Oman; 3 Dow University of Health Sciences, Karachi 74800, Pakistan; 4 Department of Radiation Oncology, Cyberknife and Tomotherapy Centre, JPMC, Karachi 75510, Pakistan; 5 Jinnah Sindh Medical University, Karachi 75510, Pakistan

**Keywords:** oncofertility, radiation, ovarian transposition, cryopreservation, tumour board

## Abstract

With reduced cancer mortality in recent years, increased efforts must be put into safeguarding cancer survivors’ long-term quality of life (QOL). Fertility preservation is recognised as a key component of QOL in survivorship. Concerns about fertility have been seen to significantly impact cancer patients’ emotional and mental health as, generally, both malignancy and its treatment may cause a temporary or permanent reduction in infertility. This article reviews the primary effects of radiation therapy on male and female gonads and has further highlighted procedures through which the functioning of these organs can be protected before or during radiation treatment. We have also emphasised the importance of the establishment of multidisciplinary tumour boards and patient education regarding future reproductive function which is an important component of the care of individuals with cancer. This article highlights that infertility is a persistent and major concern that can add to long-term stress in cancer survivors, and education about fertility preservation before the initiation of any treatment is especially important.

## Introduction

The 21st century has seen a decline in cancer mortality in the adolescent and young adult population, and this has caused the management of quality of life (QOL) to become an essential part of cancer management. Concerns about fertility outcomes considerably impact a cancer survivor’s psychological status and thus their QOL after treatment [1, 2]. Preservation of fertility thus becomes an important objective in the overall comprehensive management plan of oncological treatment, which is made all the more relevant in the recent age of decreased cancer-related mortality [3]. All malignancies (e.g. lymphoma, Wilms, sarcoma) in which radiotherapy is delivered to ilioinguinal and abdominal region or directly irradiating gonads such as acute leukaemia or gonadal tumours can have significant reproductive effects, including acute gonadal failure and infertility. Therapeutic advances in chemotherapy and radiotherapy have improved survival rates but may also permanently impact the reproductive capacity of a cancer survivor [4, 5].

As low and middle-income countries (LMICs) continue to be confronted with economic challenges and change in population dynamics, it is essential to continue adapting the most practical and cost-effective healthcare model. Estimating and minimising the risk of infertility before radiotherapy may mitigate the need for costly and invasive fertility-preserving therapies in our low and middle-income populations [6]. Investing in education and training and integration of fertility preservation into public or private health care providers should remain a top priority. The acceptableness of child adoption also differs across the different demographics of Asia [7]. The reasons listed above demonstrate the need for clinical research to educate cancer care providers regarding different fertility preservation options and their timely utilisation. Moreover, the need for the establishment of multidisciplinary tumour boards has been identified that cater to the patients’ oncofertility support needs.

## Method

We searched PubMed, Embase, ScienceDirect, Web of Science and Google Scholar (2001–2021) for studies discussing all aspects of oncofertility research and radiation practice with the goal to preserve fertility. The literature was reviewed using keywords oncofertility, radiotherapy, fertility preservation, gonadotoxicity, oncofertility model of care, LMIC, tolerance dose, toxicity as well as combination of these words to help the search The information was categorised into three major domains: radiation-induced gonadal toxicity, fertility preservation methods utilisation before and during radiotherapy and the role of multidisciplinary tumour board in LMIC. A peer review team consists of three members who have no conflict of interest, two of whom were external to the university programme but internal to the entity finalised the article to be reviewed. A summary of all the included articles is listed in Table 1.

## Radiation fractionation schedule & oncofertility

### Female

The dose at which 97.5% of the women suffer from post-treatment ovarian dysfunction immediately after treatment is known as the effective sterilisation dose (ESD) [8, 9]. It decreases with age due to the fall in ovarian reserve. Typically, the ESD is 20.3 Gy in newborns and falls to 14.3 Gy once they reach the age of 30 [10]. However, it should be kept in mind that ovarian reserve may vary considerably in women due to the effect of different genetic or environmental factors [11, 12]. The gonads are one of the most radiosensitive cells and are vulnerable to the damaging effects of radiation with reports that radiation dose of as little as ≤2 Gy can decrease the ovarian reserve of the irradiated organ by 50% [13]. The risk of infertility from radiation therapy increases with age because the ovarian reserve of an individual falls as they grow. Fertility risk associated with pelvic or whole abdominal radiation is noted to be high in doses of ≥6 Gy in adult females, ≥10 Gy in post-pubertal females and ≥15 Gy in prepubertal females [14–17]. Sterilisation by radiation to ovaries is immediate (there is no latent period, as in males). In testis, a dose of 0.1 Gy leads to temporary sterility and a dose of 6 Gy leads to permanent sterility. Fractionation of radiation dose is one of the techniques used to decrease fertility risk while targeting the destruction of neoplastic cells for improving the therapeutic index [10]. Fractionated and continuous low dose rate irradiation is more effective than a single exposure because a large proportion of stem cells are in a radio-resistant phase of the cell cycle. In studies examining the sterilisation effects of radiotherapy on women, an increased number of dose fractions showed a correlation with decreased fertility risk, e.g. a dose of 20 Gy given in 6 weeks proffered a sterilisation risk of 50% [15].

### Male

Pioneering studies conducted in the late 20th century concluded that even radiotherapeutic doses of scatter radiation impaired spermatogenesis. They also noted that smaller radiation doses have a greater effect on the function of seminiferous tubules as the cells enter a period of senescence once the radiation dose is increased beyond 6–8 Gy. Furthermore, instead of facilitating testicular function, dose fractionation seemed to harm sperm production [11, 12]. There is little effect of radiotherapy on Leydig cells so although irradiation of the testes may lead to sterility, it has little or no effect on the libido. It has also been studied that radiation doses of more than 20 Gy cause damage to these cells leading to the need for exogenous testosterone administration [18]. Recent literature review shows that in fractionated regimen, total dose of >2.5 Gy caused complete loss of testicular function, while if a single fraction is delivered, radiation doses of more than 6 Gy were needed to cause total azoospermia [19]. While the benefits of fractionated radiotherapy still require research in the preservation of fertility in males suffering from extra-testicular cancers, it has been proven to be useful in the treatment of testicular cancers as described in Table 2 [20].

The Pediatric Normal Tissue Effects in the Clinic (PENTEC) research group aims to provide clinicians best available data regarding radiation therapy and normal organ dose constraints for planning childhood cancer treatment. They reported maintaining an ovarian dose of <2 Gy to best preserve oocytes and prevent ovarian failure and doses greater than 20 Gy are expected to result in high risk (70%–100%) of acute ovarian failure and death of all oocytes in all female patients, regardless of age [21]. PENTEC also investigated that doses starting from 10 Gy almost always result in azoospermia after testicular radiation exposure [22].

## Fertility preservation & oncofertility

### Cryopreservation

Cryopreservation can be done in three parts as shown in Table 3:

#### Cryopreservation of fertilised embryo

This entails the fertilisation of mature oocytes with sperm samples via in vitro fertilization and in vitro intracytoplasmic sperm injection. Although success has been noted with this form of fertility preservation, religious, legal and ethical limitations may make it a difficult choice for many patients presenting in our socio-cultural setup but this procedure can be considered in older patients and with an established relationship [9].

#### Cryopreservation of oocytes or spermatocytes

This is an established form of fertility preservation procedure in which mature oocytes are frozen before being fertilised by a sperm. This may help overcome a few religious and ethical barriers and may also be the optimum choice for women who do not have partners at the time of treatment. One limiting factor of oocyte preservation is that it is a time-consuming process carried out in older post-pubertal patients with an emotional maturity that may not be practical in the setting of aggressive tumours [9].

Cryopreservation of spermatocytes is usually done by the preservation of ejaculate [23]. In the event of retrograde ejaculation, the use of alpha-agonists or sperm collection from urine after alkalinisation of the urine is prescribed [24]. Collection of ejaculate via stimulatory methods like masturbation (most common), penile vibratory stimulation or electroejaculation is another means of spermatocyte collection. All of the above-listed methods are only viable for post-pubertal males. Azoospermia is one of the leading causes of failure of these methods but may be misdiagnosed in some patients [25].

#### Cryopreservation of ovarian or testicular tissue

Cryopreservation of the ovarian tissue is currently the only option offered to prepubertal patients. It employs the slow-cooling of small pieces of ovarian tissue obtained via laparoscopy or laparotomy which are later transplanted into the body. Recent research has reported successful pregnancies with this procedure. However, restoration of complete endocrine function in transplanted tissue has still not been effective.

However, it is accompanied by the risk of cancer reseeding, particularly in blood-borne cancers but such studies are still underway and clinically relevant data are not yet available [26].

Cryopreservation of testicular tissue following surgical withdrawal (Testicular sperm extraction (TESE)) is done in the event of a failure in ejaculate collection. Percutaneous, open and microsurgical TESE procedures can be successfully employed in the event of failure of adequate sperm collection through the non-invasive methods listed above [27]. Microsurgical TESE is comparatively successful with a 50% pregnancy rate after a mean period of 18 years [28].

Summarised fertility preservation methods and their success rate are mentioned in Table 3.

### Ovarian transposition

This is a process that entails that the location of the ovaries in the body is changed from a normal, entopic position to a different anatomical site to protect them from the harmful effects of abdominal or pelvic radiation which are used in lymphomas, Wilm’s tumour, pelvic Ewing’s sarcoma & rhabdomyosarcoma, etc. Some anatomic placements include the area within the paracolic gutters, along the iliac crest and opposite to the radiation field site [29]. Turkgeldi *et al* [30] evaluated the effectiveness of laparoscopic ovarian transposition and ovariopexy and reported that 22 (65%) patients retained ovarian functions. A total of 12 (35%) patients that were not able to retain their fertility had chemotherapy concurrent to radiation therapy, were above 30 years of age and had cervical cancer [31]. Similar success rates of ovarian transposition were reported in a systematic review of 35 papers [31]. The risk of metastasis to ovaries has been a major concern, but the debate was resolved by a meta-analysis of 24 studies that confirmed the efficacy of ovarian transposition and proved negligible risk of metastases in the transposed ovary in the setting of radiation therapy [32]. There are certain drawbacks of the procedure including the development of ovarian cysts, adhesions, pelvic pain, ovarian migration and tubal injury. The most common position for ovarian transposition reported is paracolic gutters, contralateral to the tumour and in line with the iliac crest depending upon the type and location of the tumour. Transpositioning of ovaries to this anatomical position outside the radiation field also leads to fewer complications [33].

### Gonadal shielding

ALARA is an important principle in the field of radiation that guides the approach to the administration of radiation doses to the human body. ALARA stands for ‘as low as reasonably achievable’. This implies that if even a small portion of the radiation dose that has no direct benefit is administered, then it should be avoided. To employ this guideline, three protective measures are used to provide radiation safety: time, distance and shielding. Here, we discuss how shielding can be useful for gonadal safety and fertility preservation [34].

#### For pelvic malignancy

International Commission on Radiological Protection 34 states ‘Gonads of individuals with reproductive capability should be safeguarded if present within or at a distance of 5 cm of the principal beam, and if the shielding does not preclude major diagnostic details or impede with the investigation’ [35]. Traditionally, gonad shielding can decrease the dose to testes around 95% and ovaries around 50% but with the use of advance radiation techniques and equipment resulting in more effective control of testicular dose, shielding can be a re-considered option in the management of pelvic malignancies [27].

#### Total body irradiation (TBI)

Many paediatric genetic conditions and malignancies are managed with the help of haematopoietic stem cell transplantation (HSCT) which has led to a much-expanded population of adult survivors than it was before the use of HSCT. With this increasing survival rate, there has been a focus on the improvement of side effects and QOL changes that are associated with the treatment procedures. Total body irradiation (TBI) is an integral part of HSCT procedure in some protocols and can result in infertility as a common late effect that causes major psychosocial problems for both the patient and the family later on [25]. A single-institution study verified an 80%–85% reduction of testicular dose by using different methods of testicular shielding in patients receiving TBI [36]. In sexually mature patients, strategies that include semen cryopreservation, hormonal suppression and collection of sperm surgically can be employed but complete spermatogenesis cannot be attained in most paediatric populations by these means limiting the use of these strategies. This leads to the favouring of the use of gonadal shielding by placing lead or more commonly now with MLC which leads to a significant reduction in unnecessary gonadal radiation exposure but with limitations. Education about fertility preservation methods prior to the initiation of any cancer treatment is important because assessment of fertility potential after cancer therapy is challenging and fertility may be permanently impaired. A meta-analysis of 18 studies published in 2017 concluded that the current practice of gonadal shielding in female pelvic radiography is not considered as efficacious in the reduction of radiation exposure [37]. However, concerns of anatomical variation in the location of the ovaries in different individuals may be slightly overcome with the use of radiological investigations including ultrasound, magnetic resonance imaging and CT scan [38]. The use of shielding in pelvic malignancies is contentious and is based on the best-qualified training of radiographers for accurate positioning of the shield during radiation [37]. Another study demonstrated better gonadal shielding with the incorporation of advanced radiation techniques (helical tomography) as compared to traditional methods of radiation delivery in patients requiring TBI [39].

#### Challenges in female for external shielding

The shielding in females is less efficacious, because of the variable position of ovaries in different phases of the menstrual cycle. Accurate placement of shielding requires help from a radiologist via ultrasound or placement of markers via a minimum intervention (laparoscopic) approach to ensure that ovaries are well positioned within the shielded region. In application, it is hard to set the X-ray shield accurately. In a meta-analysis, gleaned from 19 studies, the average of correctly positioned shields was noted to be just 34% so planning, resources and expertise in a team are required to achieve the needful [37].

#### Lymphoma and testicular seminoma

Maintenance of fertility is one of the major concerns for a radiation oncologist when treating a patient of the young age group. Patients with lymphoma and seminoma in which radiotherapy is delivered to abdominal nodes or pelvis required adequate shielding of the testes to preserve testicular functions from scattered radiation. Such patients benefit from external testicular shielding made of Cerro band alloy. But it is important to have an evaluation from an endocrinologist; as most of the chemotherapy regimens used for above both diseases have deleterious effects on spermatogenesis leading to approximately 30% effect on fertility [38]. Studies conducted in patients that were treated for abdominal nodes or other pelvic fields where testicular shielding was not used and incorporation of newer radiation techniques (volumetric modulated arc therapy and intensity modulated radiotherapy) are effectively limiting the dose to gonads, making it an important discussion point to utilise external testicular shielding or not for patients with testicular tumours [39].

#### Controversies and solution

The contribution of gonadal shielding to safeguard the patient’s reproductive organs has long been argued upon without reaching an agreement, giving rise to contradictory guidelines. One drawback is that gonadal shielding only blocks direct radiation exposure, leaving the patient vulnerable to scatter radiation [41]. Regardless of the contradictions in the scientific literature on the importance of gonadal shielding still stands in above mention scenarios. The data shows that gonadal shielding is invariably utilised correctly. Frequent exposure to Megavoltage energies will kill its effectivity but with Kilo voltage energy imaging and with proper utilisation of marker placement as discussed above will lead to infrequently repeated X-ray resulting in satisfying ALARA principle [42]. It’s important to understand the challenge of low health literacy in our region resulting in high treatment abandonment. Discussion regarding fertility issues linked to treatment opens up Pandora’s box leading to further complicating this challenge. Discussion on this topic among peers can be a potential solution that will help a caregiver a chance to use the proper shielding method to opt or to negate its use [43].

### Multidisciplinary tumour board establishment for oncofertility care

Oncofertility is a comparatively new discipline that is becoming increasingly significant as light is shed on the QOL of cancer survivors. The most important aspect is the information gap due to inconsistency with which cancer patients are treated in different cancer centres of LMIC. The ability to advise patients about the impact of particular cancer treatment on fertility is important and requires an interdisciplinary team involving oncologists, oncological surgeons nurse navigators and reproductive medicine specialists which expands fertility options for cancer survivors. The close relationship of an oncofertility consortium with a cancer patient’s treatment process should be planned and considered. Studies have noted that the QOL of cancer survivors is negatively impacted by a lack of knowledge regarding their fertility outcomes. In juxtaposition, the presence of fertility counselling before initiation of treatment and continued care through and beyond the treatment process has been linked to the decreased impact of fertility concerns on the patient’s mental state, improving their QOL [44, 45]. Furthermore, it has been noted that the presence of a fertility navigator tremendously helps to address and alleviate the patients’ concerns regarding their future fertility [46].

Despite the pivotal nature of fertility discussions in the QOL of cancer survivors after tumour regression, the U.S. Quality of Oncology Practice Initiative reported only a 40% rate of fertility risk discussions during cancer management in the years 2013–2016 [47]. This discrepancy may be due to a lack of formal training or due to a lack of knowledge regarding different techniques as similar issues are also highlighted in a study from the Arab world [48]. Families dealing with cancer in our region have a low literacy rate, poverty, cancer stigma, lack of access to healthcare, lack of proper diagnostic procedures, late diagnosis, lack of cancer therapy options, inability to manage treatment toxicities and treatment abandonment. A study conducted in Pakistan found that around 90% of female cancer patients lacked awareness about the possible ramifications of treatment on fertility [49]. Another study reported that the provision of formal guidelines helps in the facilitation of discussions regarding fertility in the oncological setup [50].

**Figure 1.**
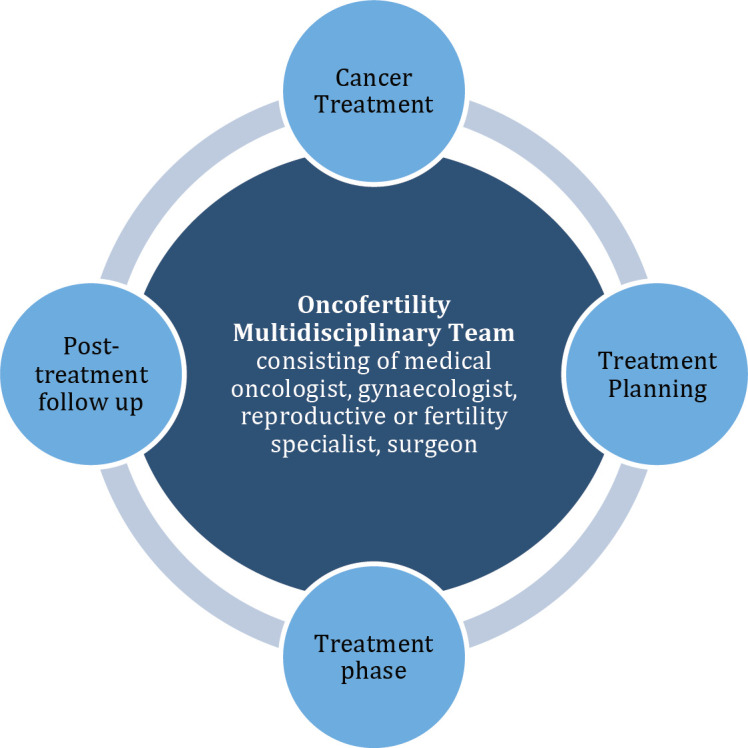
Exemplifies the close relationship of the Oncofertility team with a patient’s cancer treatment algorithm.

In our opinion, discourse must be opened regarding the critical nature of these discussions. Furthermore, as the presence of professionals from different fields is of paramount importance in ensuring the quality of care, we think it is essential to discuss all such cases in a designated fertility multi-disciplinary team board meeting so that optimum fertility care can be provided to the patients as shown in Figure 1. These oncofertility tumour boards can be established on the findings of studies that have been conducted to analyse systems of oncofertility care that are already in place. Online platforms can also be utilised to facilitate the fertility preservation process. This would be especially helpful in situations where there are issues of resource and transport allocation that limit the delivery of care.

## Conclusion

Oncofertility is a new and evolving field that can play a crucial role in determining a patient’s QOL after and during their cancer treatment. Ongoing research efforts have led to expanded fertility preservation options for both males and females while it still requires further study. This article will help establish standard procedures for discussing fertility preservation methods with cancer patients in order to fully assist cancer survivors with fertility concerns. Further research in this discipline and improvement of the quality of fertility support and care being provided to cancer patients can be done best under the shadow of a multidisciplinary team which increases the proportion of patients achieving durable disease control with acceptable functional outcomes.

## Authors’ contributions

Dr Urooba and Dr Yumna developed the concept.

Dr Hammad and Dr Fatima drafted the article.

Dr Sehrish, Dr Arham and Muhammad Hasan revised the article critically for important intellectual content.

Dr Bilal and Dr Ahmed Nadeem Abbasi finally approved the version to be published.

## Conflicts of interest and funding

No funding was received for this article and the authors report no conflicts of interest in this work.

## Figures and Tables

**Table 1.** Summary of searched articles.

Databases searched (date run: June 2010, date rerun: January 2000)				
Included reference	Format	PubMed	Google Scholar	Cochrane
Miller *et al* [1]	Jnl	x		
Benedict *et al* [2]	Jnl	x		
Harzif *et al* [3]	Jnl	x		
Kanbar *et al* [4]	Jnl	x		
Gargus *et al* [5]	Jnl	x		
Islam *et al* [6]	Jnl	x		
Adewunmi *et al* [7]	Jnl	x		
Biedka *et al* [8]	Jnl		x	
Mahajan [9]	Jnl	x		
Oktem *et al* [10]	Jnl	x		
Mariani *et al* [11]	Jnl	x		
Salama and Woodruff [12]	Jnl	x		
Terenziani *et al* [13]	Jnl	x		
Roberts *et al* [14]	Jnl	x		
Marci *et al* [15]	Jnl	x		
Rodriguez-Wallberg and Oktay [16]	Jnl	x		
Wo and Viswanathan [17]	Jnl	y		
Oktem *et al* [10]	Jnl	x		
Schuck *et al* [18]	Jnl	x		
Charles [19]	Jnl	y		
Kinsella *et al* [20]	Jnl	y		
Hill-Kayser *et al* [21]	Jnl		x	
Hoppe *et al* [22]	Jnl		x	
Stahl *et al* [23]	Jnl	x		
Abram McBride and Lipshultz [24]	Jnl	x		
Coward *et al* [25]	Jnl	x		
Sarnacki [26]	Jnl	x		
Osterberg *et al* [27]	Jnl	x		
Hsiao *et al* [28]	Jnl	x		
Duncan *et al* [29]	Jnl	x		
Turkgeldi *et al* [30]	Jnl	x		
Gavrilova-Jordan *et al* [31]	Jnl	x		
Mossa *et al* [32]	Jnl	x		
Irtan *et al* [33]	Jnl		x	
Meistrich [34]	Jnl	x		
Sage Publications Limited [35]	Book		y	
Abram McBride and Lipshultz [24]	Jnl	x		
Agarwal *et al* [36]	Jnl		x	
Karami *et al* [37]	Jnl	x		
Huddart *et al* [38]	Jnl	x		
Singhal *et al* [39]	Jnl	x		
Dibs *et al* [40]	Jnl	x		
Mazonakis *et al* [41]	Jnl	y		
McKenney *et al* [42]	Jnl	x		
Daniels and Furey [43]	Jnl	y		
Ehrbar *et al* [44]	Jnl			x
Wang *et al* [45]	Jnl	x		
van den Berg *et al* [46]	Jnl	x		
Irene *et al* [47]	Jnl	x		
Kassem *et al* [48]	Jnl	x		
Mahey *et al* [49]	Jnl	x		
Benedict *et al* [50	Jnl	x		

Codes: x = found from the search

Y = in database, found when searching strategy rerun

Format codes: jnl = journal article

**Table 2.** Dose constraints for conformal radiation planning.

Structure	Irradiation type	Endpoint	Dose (Gy)
Testes	Conventional	Temporary sterility Permanent sterility	0.1–0.15 Gy 6 Gy
Ovary	Conventional	Permanent infertility Hormone insufficiency	5–10 Gy 10–15 Gy

**Table 3.** Fertility preservation methods and their success rate.

Fertility preservation methods	Patients	Success rate
Female		
Cryopreservation of fertilised embryo	Post pubertal	59% (91) [45]
Cryopreservation of oocytes	Post pubertal	44% (93) [46]
Cryopreservation of ovarian	Prepubertal	Experimental
Male		
Cryopreservation of spermatocytes	Post pubertal	36%–50% [47]
Cryopreservation of testicular tissue	Pre pubertal (experimental) Post puberty	Experimental
